# Supplementary material for: Tumor histoculture captures the dynamic interactions between tumor and immune components in response to anti-PD1 in head and neck cancer
Source: Nat Commun. 2024 Feb 21;15:1585. doi: 10.1038/s41467-024-45723-z (PMC10881470; doi:10.1038/s41467-024-45723-z)
Supplement: Supplementary file 1 — Supplementary Information [file 41467_2024_45723_MOESM1_ESM.pdf]

Tumor histoculture captures the dynamic interactions between tumor and immune components in response to anti-PD1 in head and neck cancer

Supplementary Information

Supplementary Figure 1

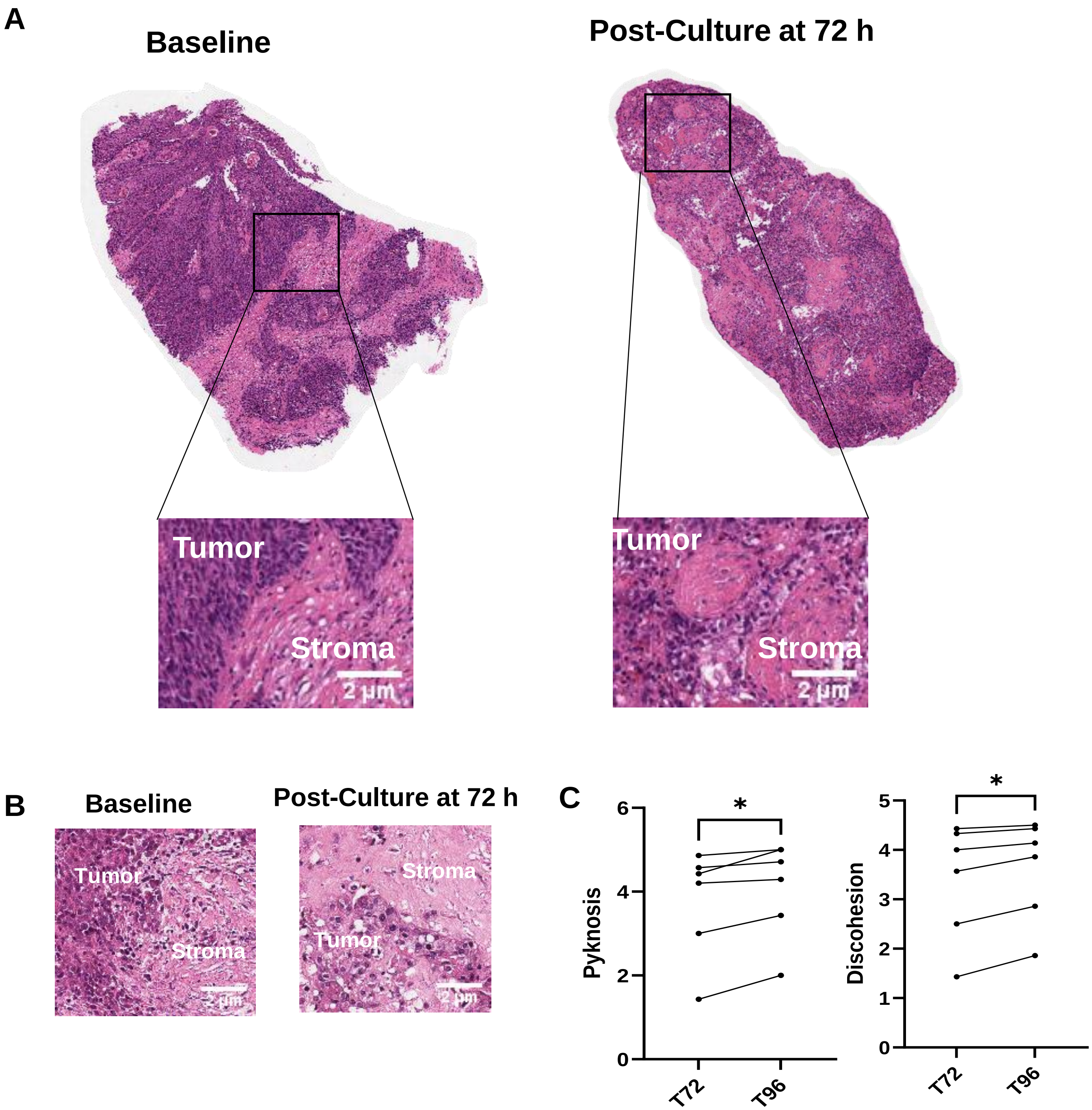

Supplementary Figure 1: Tumor architecture preservation post 72-hour culture. A: Figure is the representative image from one treatment naive sample, displaying the tumor and stromal architecture preservation post culture at 72 hours. B: Figure is the representative image from a pre-treated sample, displaying the tumor and stromal architecture preservation post culture C: Data showing increased pyknosis and discohesion at 96 hour (T96) of culture compared to 72 hours (T72) (n=6 patient samples). Data represented as symbol and line graph with paired t-test performed using Wilcoxon matched-pairs signed rank two tailed test. (*p<0.05). Source data are provided as a Source Data file.

Supplementary Figure 2

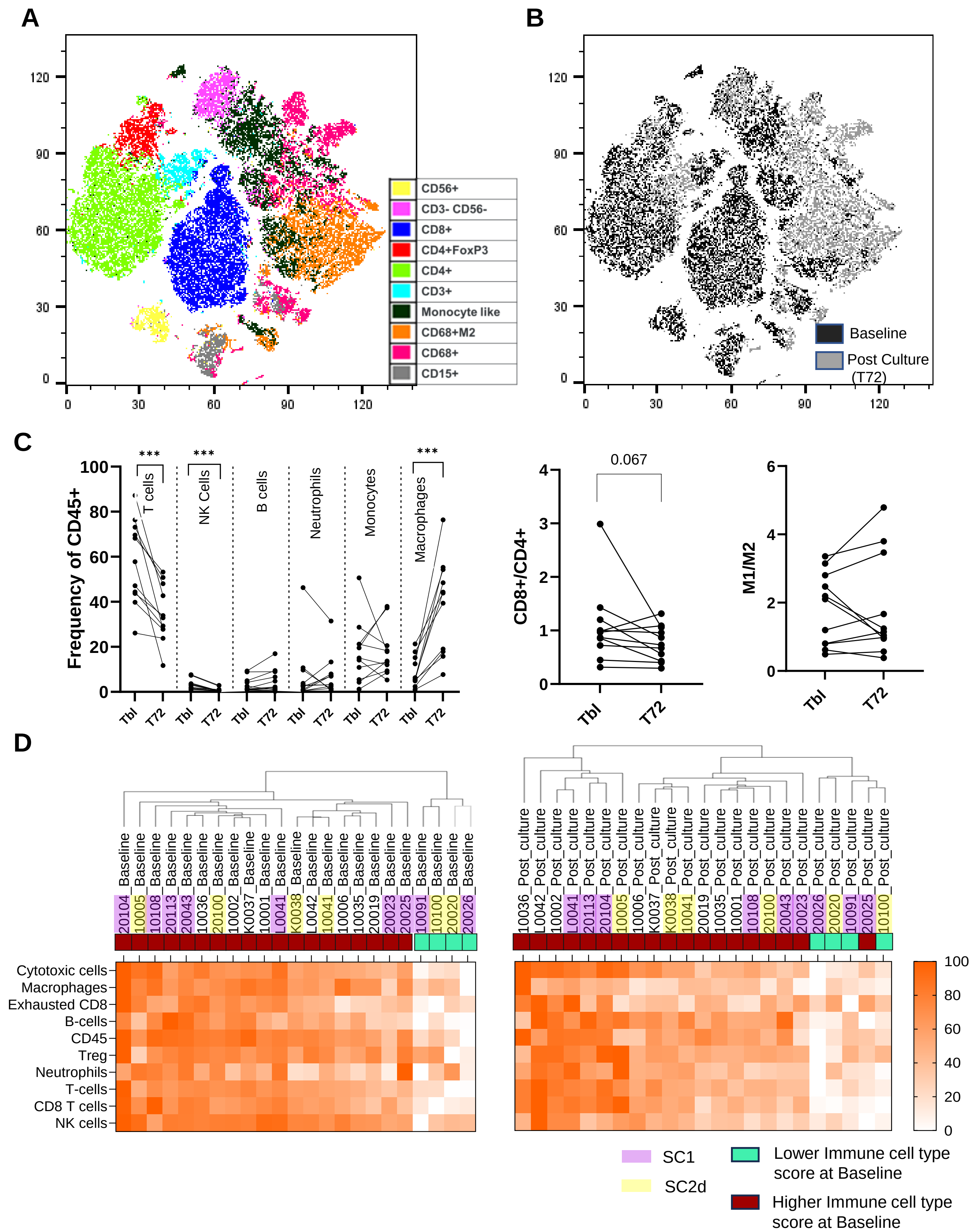

Supplementary Figure 2: Immune cell proportions in tumor microenvironment A) tSNE representation using concatenated baseline and their respective post culture data plotted for n=8 Patient samples. The various immune sub-populations evaluated using flow cytometry are highlighted using different colors. B) The tSNE plot presented in 2A with an overlay of the baseline (dark grey) and post-culture (light grey) profiles of immune sub-populations for n=8 Patient samples. C) Preservation of live immune cell type proportions at baseline and post-culture for 11 patient samples is represented. All cell types are shown as percentage of CD45. In addition, T cell (CD8+/CD4+) and Macrophage (M1/M2) sub-populations are expressed as ratios. Data is represented as symbol and line graph with paired t-test performed using Wilcoxon matched-pairs signed rank two tailed test. (***$p<0.001$). D) Level of immune cell sub-populations ("hotness") was compared at baseline and post culture using immune cell signature scores from NanoString normalized mRNA data. Heatmap representation of this data is presented along with Euclidian distance-based hierarchical clustering using data from 23 patient samples .Source data are provided as a Source Data file.

Supplementary Figure 3

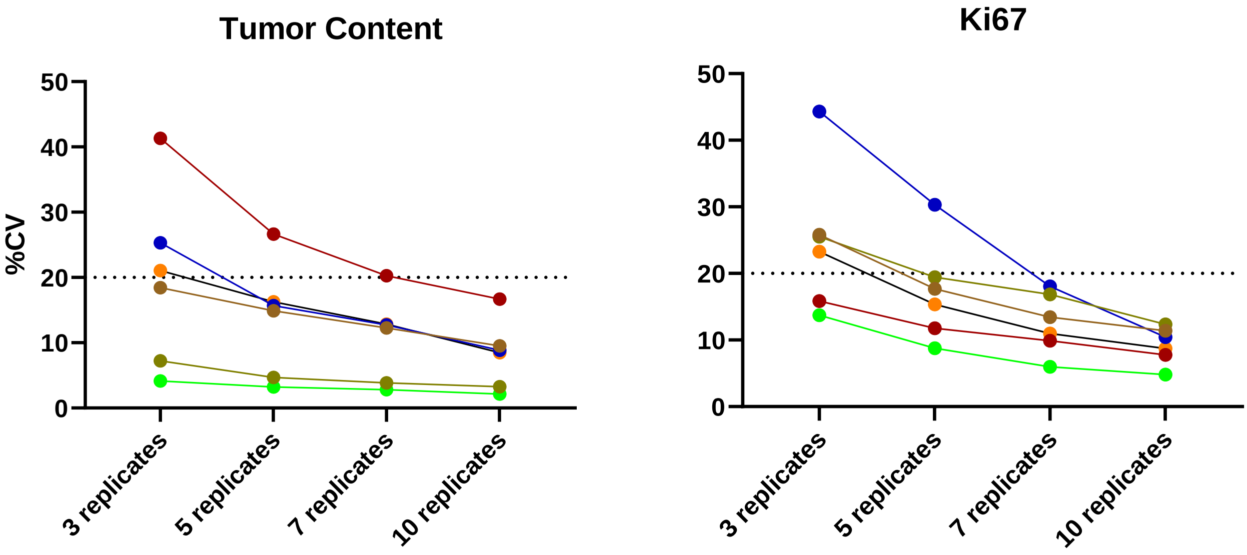

Supplementary Figure 3: Determining optimal number of replicates per arm. A) Tumor content (left) and Ki67 (right) data was evaluated for 3,5,7 and 10 replicates for 6 patient samples. Data represents the coefficient of variance (%CV) across 30 simulated arms containing different replicate numbers. Source data are provided as a Source Data file.

Supplementary Figure 4

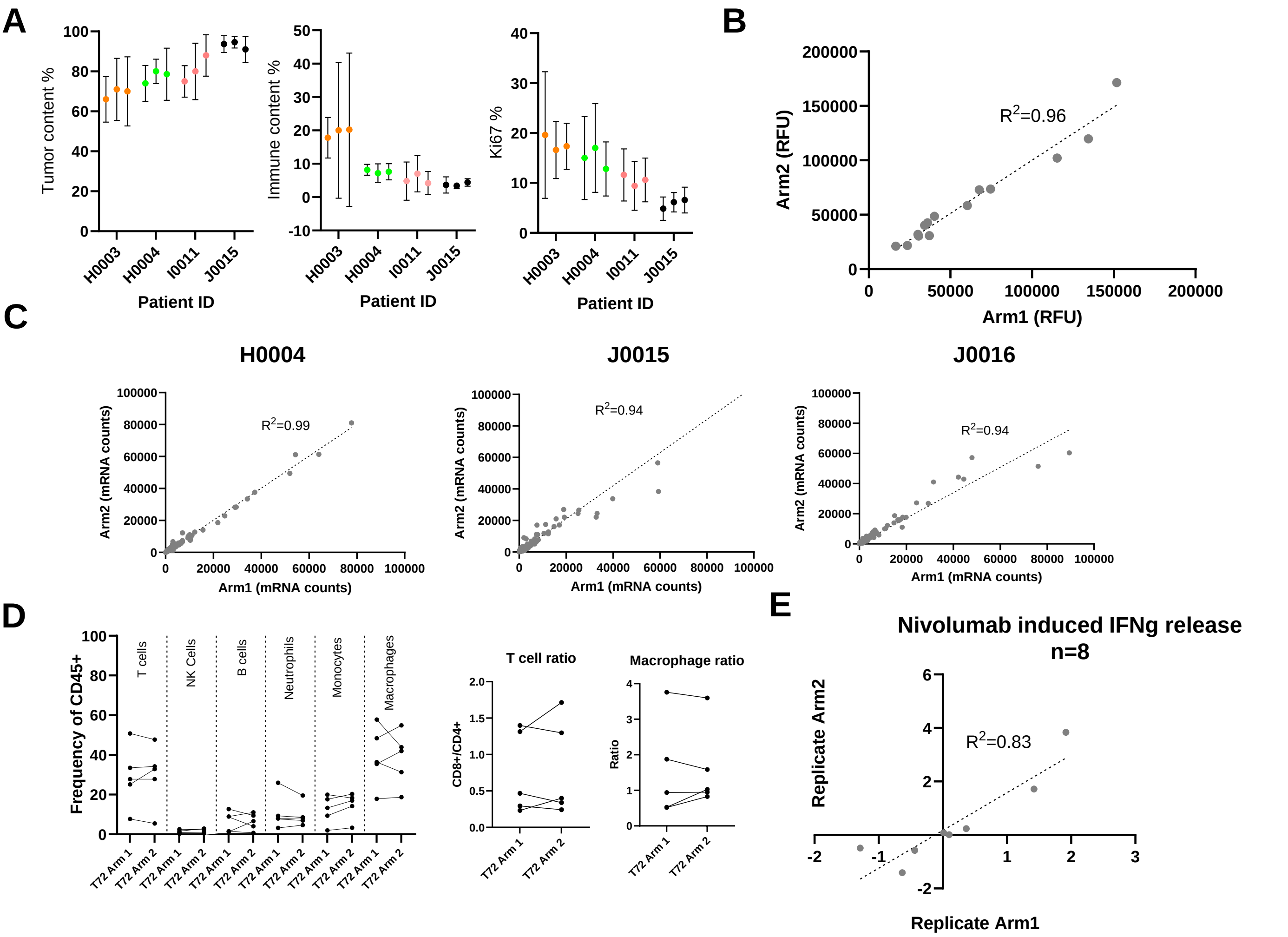

Supplementary Figure 4: Establishing arm equivalence and reproducibility across arms of TruTumor platform. A) Tumor content, immune content and Ki67 expression in tumor was assessed across arms from H&E and IHC slides by pathologists at baseline. Data (n=4 Patients sample) is represented as Mean±SEM and p-value significance was evaluated using one-way Anova for each sample using Tukey's multiple comparison test. B) PrestoBlue assay was used to assess the viability of duplicate arms. Data is represented as the average relative fluorescence unit (RFU) of all the fragments in each arm at baseline across 14 patient samples. Simple regression analysis was performed and R^2 values are denoted. C) Gene expression assessed by normalized mRNA counts generated from NanoString assay was performed on duplicate arms at baseline for three patient samples. Simple regression analysis was performed and R^2 values are denoted. D) Immune sub-populations was analyzed using flow cytometry on duplicate arms, 72 hours post culture, and represented as percentage of CD45 (n=5 Patient samples). Additionally, T cell and Macrophage sub-populations are expressed as ratios. Data is represented as symbol and line plot and paired t-test being was performed using Wilcoxon matched-pairs signed rank two tailed test. E) Nivolumab treatment response was evaluated by IFNg release in duplicate arms from each sample. Data is represented as Log2 fold change with respect to control for 8 patient samples. Simple regression analysis was performed and R^2 values are denoted. Source data are provided as a Source Data file.

Supplementary Figure 5

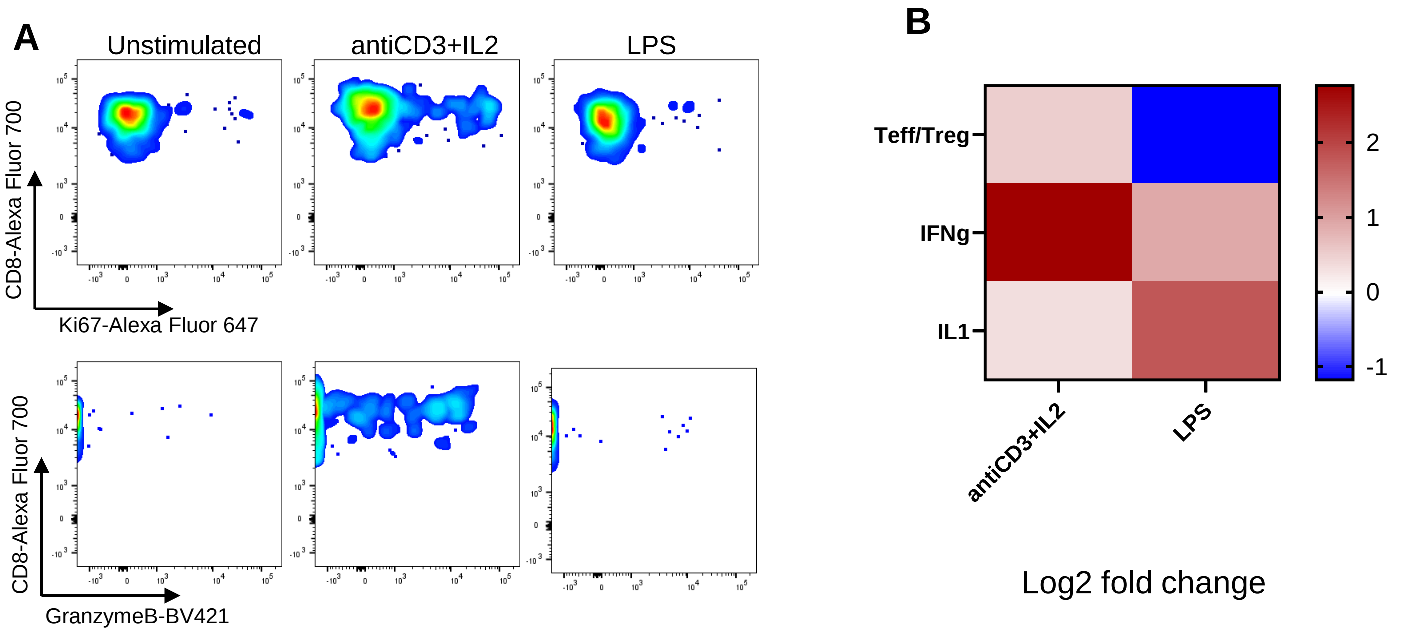

Supplementary Figure 5: Evaluating Immune cell activity in TruTumor platform.
A) Representative flowcytometry data of a sample shown demonstrating proliferation of CD8+ cells (top) and increase in functional CD8+ cells (bottom) upon treatment with anti-CD3+IL2 and LPS. B) Log2 fold change in gene expression signature for CTL/Treg, IFNG and IL1 (IL1α + IL1β) with respect to control, upon treatment with anti-CD3+IL2 and LPS from a representative sample. Source data are provided as a Source Data file.

Supplementary Figure 6

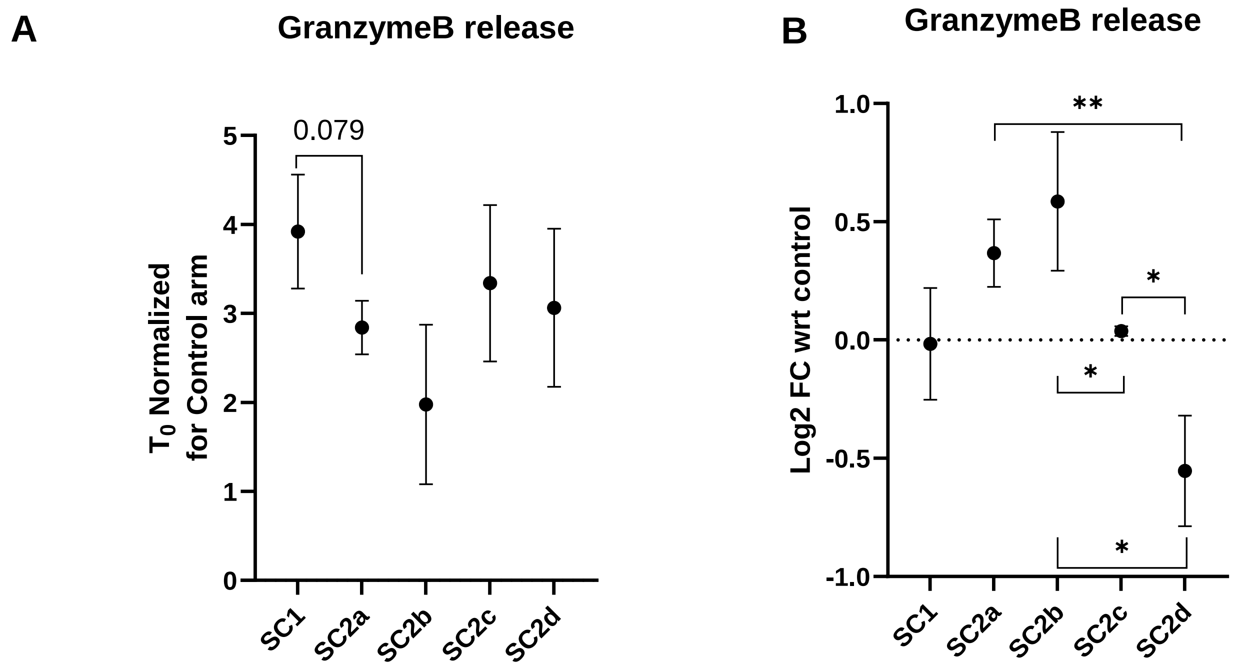

Supplementary Figure 6: GranzymeB release across various sub-cohorts (SC1:n=9, SC2a:n=27, SC2b:n=8, SC2c:n=5 and SC2d:n=6 Patient samples). A) GranzymeB release in control arm normalized to their respective T_0 (start of culture) values. Data represents T_0 normalized cytokine release as Mean ±SEM. B) Log2 fold change of GranzymeB release in Nivolumab treated arm with respect to control. Data is represented as Mean ±SEM. P-values were calculated using unpaired non-parametric Mann Whitney two tailed test. Source data are provided as a Source Data file.

Supplementary Figure 7

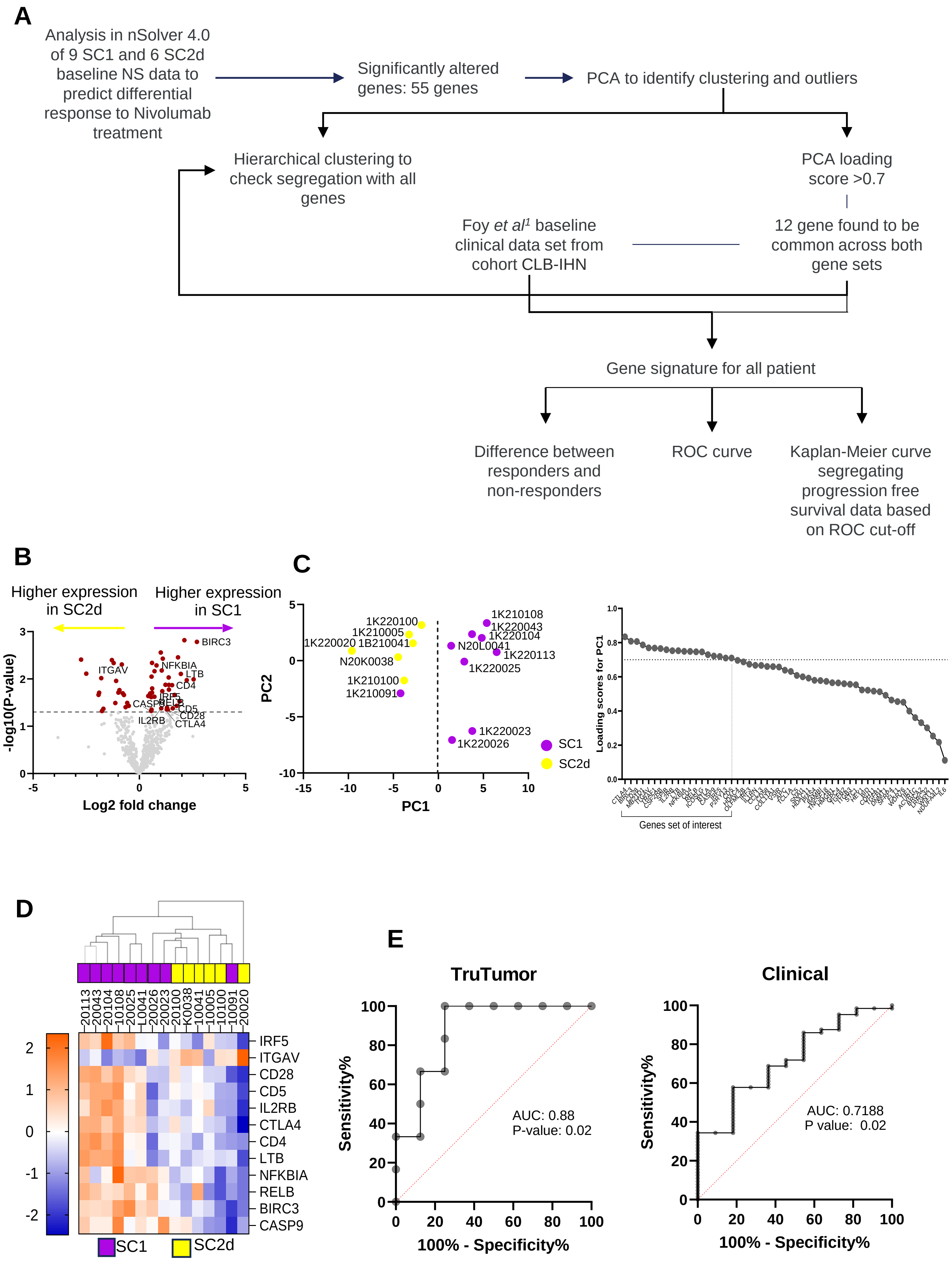

Supplementary Figure 7: Development of baseline Gene Expression Signature (GES) A) Workflow for shortlisting gene set for prediction of Nivolumab response from TruTumor platform (n=15 Patient samples) at baseline. B) Differential gene expression between SC1(n=9 Patient samples) and SC2d (n=6 Patient samples) is represented as volcano plot. Genes that show significant (Wald test, $p<0.05$) differential expression are marked in red. Genes belonging to the baseline GES are labelled. C) Left panel depicts PCA plot of 55 significantly altered genes between SC1 (n=9 Patient samples) and SC2d (n=6 Patient samples). Right panel shows knee plot of loading scores vs genes for PC1. Cutoff for loading score was kept at 0.7 (denoted by dotted line). D) Heat map representation of gene expression data for 12 genes constituting Baseline GES across SC1 (n=9 Patient samples) and SC2d (n=6 Patient samples) samples. Z-scores of GES are shown along with Euclidian distance based hierarchical clustering. E) ROC curve for the Baseline GES generated using TruTumor (n = 55 Patient samples) and published clinical anti-PD1 response data Foy *et al*[1] (n = 75 Patient samples). Source data are provided as a Source Data file.

Supplementary Figure 8

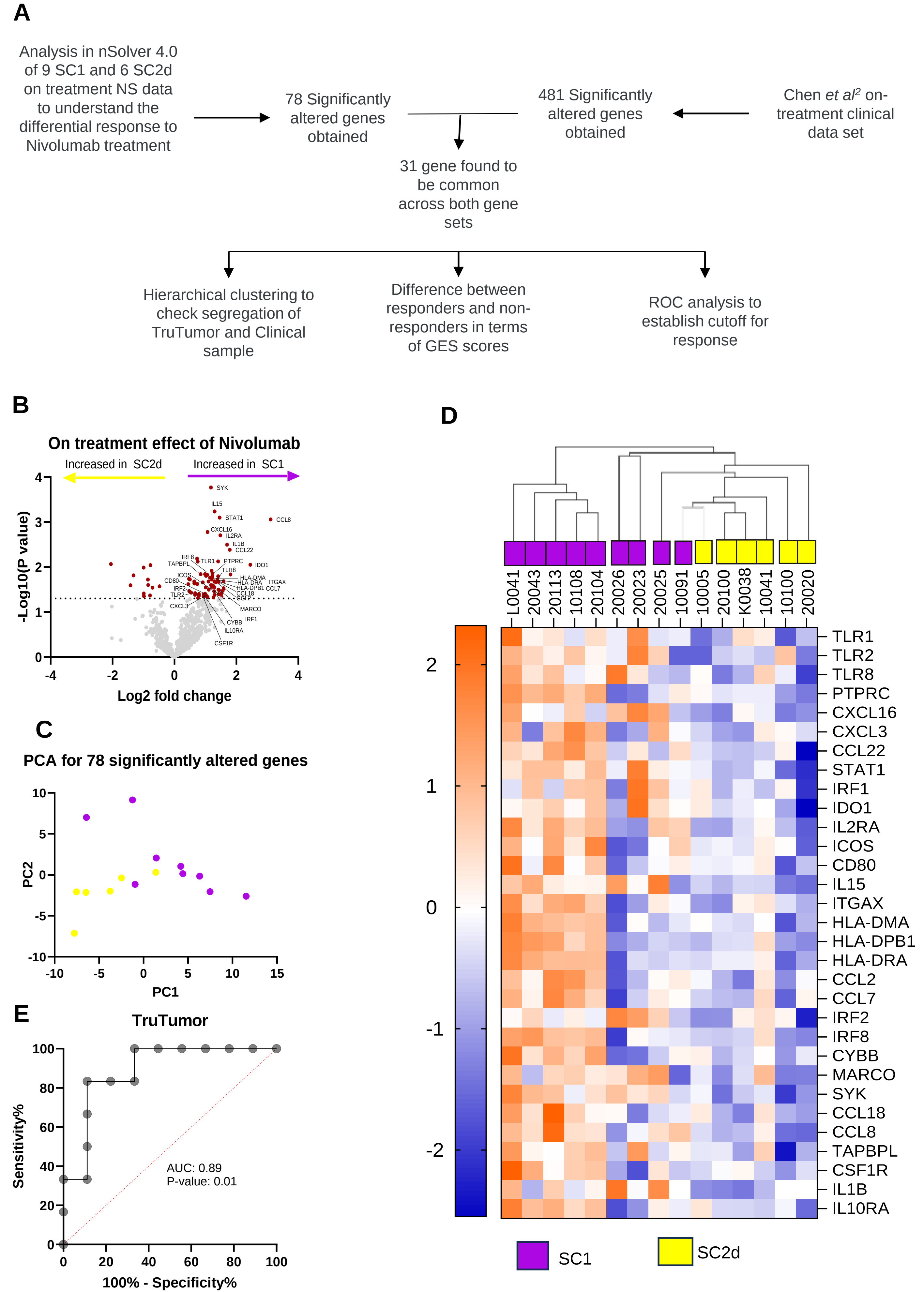

Supplementary Figure 8: Development of on-treatment Gene Expression Signature (GES) : A) Workflow for shortlisting gene set for prediction of Nivolumab response from TruTumor platform (n=15 Patient samples) on-treatment chen *et al* [2]. B) Differential gene expression between SC1 (n=9 Patient samples) and SC2d (n=6 Patient samples) is represented as volcano plot. Genes that show significant (Wald test, $p<0.05$) differential expression are marked in red and 31 genes that are part of the on-treatment GES are labeled. C) PCA plot depiction of 78 significantly altered genes between SC1 (n=9 Patient samples) and SC2d (n=6 Patient samples). D) Heat map representation of gene expression data for the 31 genes of on-treatment GES across 15 SC1 (n=9 Patient samples) and SC2d (n=6 Patient samples) samples. Data is shown as as z-scores along with Euclidian distance based hierarchical clustering. E) ROC curve for the on-treatment GES generated using TruTumor. Source data are provided as a Source Data file.

Supplementary Figure 9

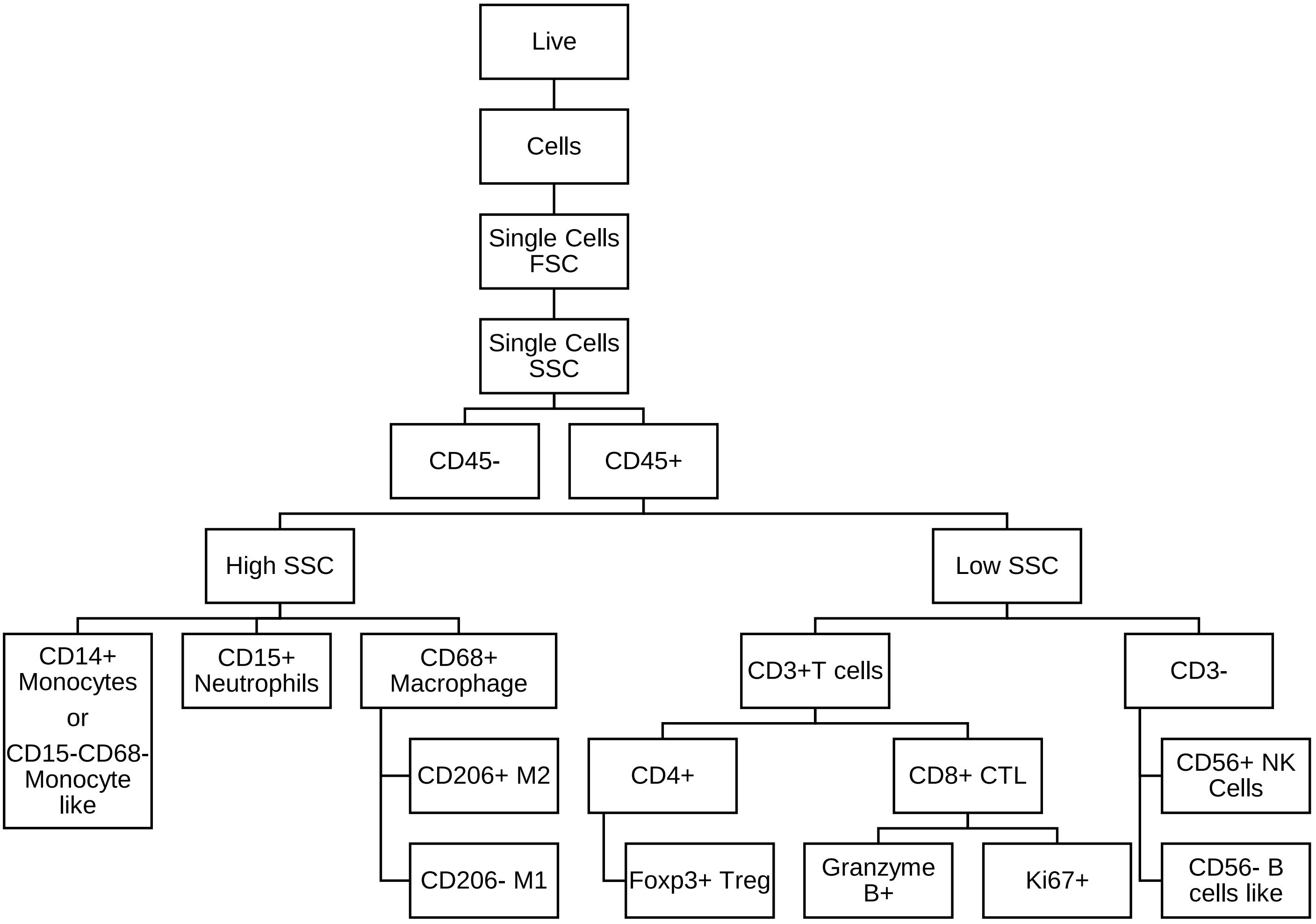

Supplementary Figure 9: Gating strategy for flowcytometry data. Cells were first gated for live population based on Live/Dead dye. Then cells were gated on forward (FSC-A) and side (SSC-A), followed by gating on single cells (FSC-H, FSC-W and SSC-H, SSC-W). Next, the cells were gated for CD45+ and then for High SSC and Low SSC population. Low SSC population was further gated for CD3+ (Pan T cells), CD4 (T helper cells), CD4+Foxp3+ (Tregs), CD8+ (CTLs), CD8+Ki67+ (Proliferating CTLs), CD8+GranzymeB+ (Functional CTLs). CD3- cells were gated for CD56+ (NK cells) and CD56- (B cell like). The High SSC population was gated for CD15 (Neutrophils), CD68+ (Macrophages), CD68+CD206+ (M2 Macrophage), CD68+CD206- (M1 Macrophage), CD14+(Monocytes), CD15-CD68-(Monocyte like).

Supplementary Table 1: Gene expression signature details

Gene Expression Signature	Genes	Reference
**TIS**	CCL5, CD27, CD274, CD276, CD8A, CMKLR1, CXCL9, CXCR6, HLA-DQA1, HLA-DRB1, HLA-E, IDO1, LAG3, NKG7, PDCD1LG2, PSMB10, STAT1, TIGIT	Ayers *et al*[3]
**IFNG**	CD3D, IDO1, CD3E, CCL5, GZMK, CD2, HLA-DRA, CXCL13, IL2RG, NKG7, HLA-E, CXCR6, LAG3, CXCL10, STAT1, GZMB, CD274, CXCL9, IFNG, LAG3	Jorgovanovic *et al*[4]
**M1**	FCGR1A CD86, CXCL10, CXCL9, IL1B, IL6, CXCL11, TNF, GBP1, CCL5, JAK2, IL1A, STAT1, IRF7, CCND1, IRF8, IRF5	NanoString nsolver Annotation
**M2**	CCL22, CCL18, CD209, MYC, CCL13, JAK2, CCND1, CD163	NanoString nsolver Annotation
**NK cell activity**	CD96, GNLY, GZMA, GZMB, GZMH, GZMK, GZMM, IFI16, IFI35, IFIH1, IFIT2, IFITM2, KIR2DL3, KIR3DL1, KIR3DL2, KLRB1, KLRD1, KLRK1, MICB, NCAM1, NCR1, NKG7, PRF1, PVR, SELL, SELP, SLAMF7, TIGIT	NanoString nsolver Annotation
**CTL**	CD8A, CD8B, CTSW, GNLY, GZMA, GZMB, GZMH, GZMK, GZMM, PRF1, RUNX3	NanoString nsolver Annotation
**Treg**	CTLA4, FOXP3, IL10, IL2RA, ITGAE, RORC, TBX21, TGFB1	Modified NanoString nsolver Annotation
**Th1**	TBX21, CASP1, CCL4, CCR2, CCR5, CD38, CSF2, CTLA4, CXCR3, IFNG, NFKBIA, IL12RB2, PRF1, CCL5, SPP1, STAT1, STAT4, TNF	NanoString nsolver Annotation
**Effector_memory**	CD70, CEBPB, CXCR6, EOMES, GZMA, HLA-DPB1, HLA-DQA1, HLA-DRA, TBX21, CXCR3	NanoString nsolver Annotation
**NK cytotoxic cells**	IL21R, KIR2DL3, KIR3DL1, KIR3DL2, NCR1, KLRB1, KLRD1, KLRK1, NKG7, CTSW, GNLY, GZMA, GZMB, PRF1, RUNX3	NanoString nsolver Annotation
**Angiogenesis**	ANGPT1, ANGPT2, ANGPTL4, CCND2, CCNE1, CES3, DLL4, E2F3, EDN1, EZH2, FGF18, FGFR1, FLT1, FSTL3, HEY1, ITGAV, ITPK1, JAG1, MMP9, MMRN2, NFIL3, PDGFB, PGPEP1, RPL7A, SERPINB5, SERPINH1, STC1, THBS1, TNFAIP6, TPM1, TYMP, VCAN, VEGFA, VEGFB, VEGFC	NanoString nsolver Annotation

**Hypoxia**	AKT1, ALDOA, ANGPT1, ANGPT2, BCL2, CDKN1A, CYBB, EDN1, EGF, EGFR, EIF4EBP1, ENO1, ERBB2, FLT1, HIF1A, HK1, HK2, IFNG, IFNGR1, IFNGR2, IL6, IL6R, LDHA, MTOR, NFKB1, NOS2, PDK1, PFKFB3, PIK3CA, PIK3CD, PIK3CG, PIK3R1, PIK3R2, PIK3R5, PRKCA, RELA, RPS6KB1, SLC2A1, STAT3, TFRC, TLR4, VEGFA, VHL	NanoString nsolver Annotation
**Matrix Remodeling and Metastasis**	A2M, BMP2, CASP3, CD36, CD44, CD47, CDH1, COL11A1, COL11A2, COL17A1, COL4A5, COL5A1, COL6A3, COMP, CTSS, ICAM1, ICAM2, ICAM3, ITGA1, ITGA2, ITGA4, ITGA6, ITGAE, ITGAL, ITGAM, ITGAV, ITGAX, ITGB2, ITGB3, ITGB8, KDR, LAMA1, LAMB3, LAMC2, LOXL2, LTBP1, MMP1, MMP7, MMP9, NCAM1, NID2, PDGFA, PDGFB, PECAM1, PLOD2, PRKCA, RELN, SERPINH1, SPP1, TGFB1, TGFB2, TGFB3, THBS1, VCAM1, VCAN	NanoString nsolver Annotation
**TGF-beta Signaling**	ACVR1C , BAMBI, BMP2, CDKN2B, ID4, IFNG, INHBA, LTBP1, MYC, RBL2, ROCK1, RPS6KB1, SMAD5, TGFB1, TGFB2, TGFB3, TGFBR1, TGFBR2, THBS1, TNF, UBB	NanoString nsolver Annotation
**Wnt Signaling**	APC, AXIN1, BAMBI, CCND1, CCND2, CCND3, CTNNB1, DKK1, FOSL1, FZD8, FZD9, GPC4, MAP3K7, MAPK10, MMP7, MYC, NFATC2, PRKACB, PRKCA, SFRP1, SFRP4, SOX11, SOX2, TP53, WNT10A, WNT11, WNT2, WNT2B, WNT3A, WNT4, WNT5A, WNT5B, WNT7B	NanoString nsolver Annotation
**Notch Signaling**	APH1B, CCND1, DLL1, DLL4, DTX3L, DTX4, E2F3, GZMB, HDAC11, HDAC3, HDAC4, HDAC5, HES1, HEY1, HIF1A, JAG1, JAG2, KAT2B, MAML2, MFNG, MYC, NOTCH1, NOTCH2, TP53	NanoString nsolver Annotation
**MAPK**	AKT1, ANGPT1, ANGPT2, BAD, BCL2L1, BLK, CASP3, CCR4, CD14, CD19, CD1C, CD2, CD27, CD274, CD28, CD38, CD3D, CD3E, CD3G, CD40, CD40LG, CD48, CD5, CD6, CD7, CD70, CD79A, CD79B, CD80, CD86, CD8A, CD8B, CD96, CHUK, CSF1, CSF1R, CTLA4, CX3CL1, CXCL10, CXCL11, CXCL13, CXCL16, CXCL9, CXCR3, DPP4, DUSP1, DUSP2, DUSP5, EGF, EGFR, EGR1, EOMES, F2RL1, FAS, FASLG, FGF13,	NanoString nsolver Annotation

	FGF18, FGF9, FGFR1, FLNB, FLT1, GNG4, GNLY, GZMA, GZMB, GZMH, GZMK, GZMM, HLA-DOB, HRAS, ICOS, ICOSLG, IDO1, IFI27, IFIT1, IFITM1, IFNG, IGF2R, IKBKB, IKBKG, IL11, IL12RB2, IL18R1, IL1A, IL1B, IL1R2, IL2RG, IRF4, IRF9, ISG15, ITGA1, JAK1, JAK2, KDR, KIR2DL3, KIR3DL1, KIR3DL2, KIT, KLRB1, KLRD1, KLRK1, KRAS, LAG3, LCK, LY9, MAP3K12, MAP3K5, MAP3K7, MAP3K8, MAPK10, MET, MS4A1, MX1, MYC, NF1, NFKB1, NFKB2, NGFR, NRAS, PDCD1, PDGFA, PDGFB, PDGFRB, PIK3CA, PIK3CD, PIK3CG, PIK3R1, PIK3R2, PIK3R5, PLA1A, PLA2G2A, PRF1, PRKACB, PRKCA, PTPN11, PVR, RASAL1, RASGRF1, RELA, RELB, SHC2, SLAMF7, STAT1, STAT2, TBX21, TGFB1, TGFB2, TGFB3, TGFBR1, TGFBR2, TIGIT, TNF, TNFRSF1A, TNFRSF25, TP53, VEGFA, VEGFB, VEGFC, ZAP70	
**PI3K-Akt**	AKT1, ANGPT1, ANGPT2, BAD, BCL2, BCL2L1, BRCA1, CASP9, CCND1, CCND2, CCND3, CCNE1, CD19, CDK2, CDK6, CDKN1A, CHUK, COL4A5, COL6A3, COMP, CSF1, CSF1R, CSF3, CSF3R, EGF, EGFR, EIF4EBP1, FASLG, FGF13, FGF18, FGF9, FGFR1, FLT1, GHR, GNG4, HRAS, IFNA1, IFNAR1, IKBKB, IKBKG, IL2, IL2RA, IL2RB, IL2RG, IL4, IL6, IL6R, IL7R, ITGA1, ITGA2, ITGA4, ITGA6, ITGAV, ITGB3, ITGB8, JAK1, JAK2, JAK3, KDR, KIT, KRAS, LAMA1, LAMB3, LAMC2, MET, MTOR, MYC, NFKB1, NGFR, NRAS, PCK2, PDGFA, PDGFB, PDGFRB, PIK3CA, PIK3CD, PIK3CG, PIK3R1, PIK3R2, PIK3R5, PRKAA2, PRKCA, PRLR, PTEN, RBL2, RELA, RELN, RPS6KB1, RPTOR, SGK1, SPP1, SYK, TCL1A, THBS1, TLR2, TLR4, TP53, VEGFA, VEGFB, VEGFC	NanoString nsolver Annotation

**Anti – Tumor Immune Activity (https://nanostring.com/research-focus/gene-expression-profiling-gep-signatures/)**		
**Gene Expression Signature**	**Genes**	**Reference**

**Cytokine and Chemokine Signaling**	AKT1, CCL13, CCL14, CCL18, CCL19, CCL2, CCL20, CCL21, CCL22, CCL3/L1, CCL4, CCL5, CCL7, CCL8, CCR2, CCR4, CCR5, CHUK, CSF1, CSF1R, CSF2, CSF2RB, CSF3, CSF3R, CX3CL1, CX3CR1, CXCL1, CXCL10, CXCL11, CXCL12, CXCL13, CXCL14, CXCL16, CXCL2, CXCL3, CXCL5, CXCL6, CXCL8, CXCL9, CXCR2, CXCR3, CXCR4, CXCR6, GNG4, HCK, HRAS, IKBKB, IKBKG, IL10, IL10RA, IL11, IL11RA, IL12RB2, IL15, IL16, IL17A, IL18, IL18R1, IL1A, IL1B, IL1R2, IL1RN, IL2, IL21R, IL22RA1, IL24, IL2RA, IL2RB, IL2RG, IL32, IL33, IL34, IL4, IL6, IL6R, IL7R, JAK2, JAK3, KRAS, NFKB1, NFKBIA, NRAS, PF4, PIK3CA, PIK3CD, PIK3CG, PIK3R1, PIK3R2, PIK3R5, PRKACB, RELA, ROCK1, SHC2, STAT1, STAT2, STAT3	NanoString nsolver Annotation
**Costimulatory Signaling**	ADORA2A, AKT1, BTLA, CD2, CD247, CD27, CD274, CD28, CD3D, CD3E, CD3G, CD4, CD40, CD40LG, CD44, CD48, CD69, CD70, CD80, CD86, CHUK, CTLA4, DPP4, EGR1, FYN, HAVCR2, HLA-DPA1, HLA-DPB1, HLA-DQA1, HLA-DQA2, HLA-DQB1, HLA-DRA, HLA-DRB1, HLA-DRB5, ICOS, ICOSLG, IKBKB, IKBKG, IL15, IL17A, IL18, IL18R1, IL2, IL2RA, IL2RB, IL2RG, IL4, LAG3, LCK, LILRB2, LY9, MAP3K7, MAP3K8, MTOR, NECTIN2, NFATC2, NFKB1, NFKBIA, PDCD1, PDCD1LG2, PIK3CA, PIK3R1, PIK3R2, PRR5, PSMB10, PSMB5, PSMB8, PSMB9, PSMC4, PTEN, PTGS2, PTPN11, PTPRC, PVRIG, RELA, RICTOR, RIPK2, SPP1, STAT4, TIGIT, TNFRSF14, TNFRSF25, TNFSF9, TRAT1, TSLP, UBB, VTCN1, ZAP70	NanoString nsolver Annotation
**Immune Cell Adhesion and Migration**	CD2, CD274, CD276, CD28, CD4, CD40, CD40LG, CD58, CD6, CD80, CD86, CD8A, CD8B, CDH1, CDH2, CDH5, CLEC14A, CLEC4E, CLEC5A, CLEC7A, CLECL1, CTLA4, CTNNB1, CXCL12, CXCR4, CYBB, HLA-A, HLA-B, HLA-C, HLA-DMA, HLA-DMB, HLA-DOA, HLA-DOB, HLA-DPA1, HLA-DPB1, HLA-DQA1, HLA-DQA2, HLA-DQB1, HLA-DRA, HLA-DRB1, HLA-DRB5, HLA-E, HLA-F, ICAM1, ICAM2, ICAM3, ICAM5, ICOS, ICOSLG, ITGA1, ITGA2, ITGA4, ITGA6, ITGAE,	NanoString nsolver Annotation

	ITGAL, ITGAM, ITGAV, ITGAX, ITGB2, ITGB3, ITGB8, MMP9, NCAM1, NECTIN1, NECTIN2, PDCD1, PDCD1LG2, PECAM1, PIK3CA, PIK3CD, PIK3CG, PIK3R1, PIK3R2, PIK3R5, PRKCA, PTPN11, PTPRC, PVR, ROCK1, SELE, SELL, SELP, SIGLEC1, THY1, TIGIT, VCAM1, VCAN, VTCN1	
**Lymphoid Compartment**	BLK, CCR4, CD19, CD1C, CD2, CD27, CD274, CD28, CD38, CD3D, CD3E, CD3G, CD40, CD40LG, CD48, CD5, CD6, CD7, CD70, CD79A, CD79B, CD80, CD86, CD8A, CD8B, CD96, CTLA4, CX3CL1, CXCL10, CXCL11, CXCL13, CXCL16, CXCL9, CXCR3, DPP4, EGR1, EOMES, F2RL1, GNLY, GZMA, GZMB, GZMH, GZMK, GZMM, HLA-DOB, ICOS, ICOSLG, IDO1, IFI27, IFIT1, IFITM1, IFNG, IGF2R, IL11, IL12RB2, IL18R1, IL2RG, IRF4, IRF9, ISG15, ITGA1, JAK1, JAK2, KIR2DL3, KIR3DL1, KIR3DL2, KLRB1, KLRD1, KLRK1, LAG3, LCK, LY9, MS4A1, MX1, PDCD1, PRF1, PVR, SLAMF7, STAT1, STAT2, TBX21, TIGIT, TNFRSF25, ZAP70	NanoString nsolver Annotation
**Myeloid Compartment**	ANGPT1, AREG, ARG1, C5AR1, CCL2, CCL20, CCL4, CCL5, CCL8, CD14, CD47, CDKN1A, CEBPB, CLEC4E, CLEC5A, CLEC7A, COL11A1, COL17A1, CRABP2, CSF1, CSF1R, CSF3R, CXCL1, CXCL12, CXCL2, CXCL3, CXCL5, CXCL6, CYBB, DAB2, DLL4, FCAR, FCGR1A, FCN1, FOSL1, FPR1, FPR3, HCK, IER3, IL1A, IL1B, IL1RN, ITGAM, ITGAX, LAMB3, LIF, LILRA5, LILRB2, LY96, LYZ, MARCO, MMP1, MRC1, NFAM1, NLRP3, NOS2, OLR1, P2RY13, PDZK1IP1, PTGS2, S100A12, S100A8, S100A9, SERPINA1, SIGLEC8, SIRPA, SIRPB2, SLC11A1, TLR1, TLR2, TLR4, TLR8, TNFAIP6, TREM1, TREM2	NanoString nsolver Annotation
**Antigen Presentation**	ATF3, B2M, BATF3, CCL4, CCR5, CD1C, CD36, CD4, CD74, CD8A, CD8B, CDC20, CTSS, CTSW, CXCL1, CYBB, DTX3L, FCGR1A, HLA-A, HLA-B, HLA-C, HLA-DMA, HLA-DMB, HLA-DOA, HLA-DOB, HLA-DPA1, HLA-DPB1, HLA-DQA1, HLA-DQA2, HLA-DQB1, HLA-DRA, HLA-DRB1, HLA-DRB5, HLA-E, HLA-F, IFNG, IRF8,	NanoString nsolver Annotation

	ITGAV, KIF2C, KIR2DL3, KIR3DL1, KIR3DL2, KLRD1, MRC1, PSMB10, PSMB5, PSMB8, PSMB9, SOCS1, TAP1, TAP2, TAPBP, THBD, TNF, TRIM21, UBA7, UBE2C, ULBP2, VHL	
**Interferon Signaling**	B2M, CD44, EGR1, EIF2AK2, FCGR1A, FLNB, GBP1, GBP2, GBP4, GHR, HLA-A, HLA-B, HLA-C, HLA-DPA1, HLA-DPB1, HLA-DQA1, HLA-DQA2, HLA-DQB1, HLA-DRA, HLA-DRB1, HLA-DRB5, HLA-E, HLA-F, ICAM1, IFI16, IFI27, IFI35, IFI6, IFIH1, IFIT1, IFIT2, IFIT3, IFITM1, IFITM2, IFNA1, IFNAR1, IFNG, IFNGR1, IFNGR2, IGF2R, IRF1, IRF2, IRF3, IRF4, IRF5, IRF7, IRF8, IRF9, ISG15, JAK1, JAK2, MX1, NCAM1, OAS1, OAS2, OAS3, OASL, PSMB8, PTPN11, RSAD2, SOCS1, STAT1, STAT2, TRIM21, UBA7, VCAM1	NanoString nsolver Annotation
**JAK-STAT Signaling**	AKT1, BCL2, BCL2L1, CCND1, CCND2, CCND3, CDKN1A, CNTFR, CSF2, CSF2RB, CSF3, CSF3R, GHR, HRAS, IFNA1, IFNAR1, IFNG, IFNGR1, IFNGR2, IL10, IL10RA, IL11, IL11RA, IL12RB2, IL15, IL2, IL21R, IL22RA1, IL24, IL2RA, IL2RB, IL2RG, IL4, IL6, IL6R, IL7R, IRF9, JAK1, JAK2, JAK3, LIF, MTOR, MYC, PIAS4, PIK3CA, PIK3CD, PIK3CG, PIK3R1, PIK3R2, PIK3R5, PRLR, PTPN11, SOCS1, STAT1, STAT2, STAT3, STAT4, TSLP	NanoString nsolver Annotation
**NF-kappaB Signaling**	BIRC3, CD27, CD40, CD40LG, CD70, CHUK, FASLG, IKBKB, IKBKG, LTB, NFKB1, NFKB2, NFKBIA, NFKBIE, PSMB10, PSMB5, PSMB8, PSMB9, RELA, RELB, RELN, TNF, TNFRSF11A, TNFRSF11B, TNFRSF14, TNFRSF17, TNFRSF18, TNFRSF1A, TNFRSF1B, TNFRSF25, TNFRSF4, TNFSF12, TNFSF13, TNFSF13B, TNFSF18, TNFSF4	NanoString nsolver Annotation
**Cytotoxicity**	BBC3, CBLC, CD47, CNTFR, FASLG, GHR, GNLY, GZMA, GZMB, GZMH, GZMK, GZMM, IFI16, IFI27, IFI35, IFI6, IFIH1, IFIT1, IFIT2, IFIT3, IFITM1, IFITM2, IFNG, IGF2R, IL11RA, IL12RB2, IL22RA1, IRF1, IRF4, IRF9, ISG15, JAK1, JAK2, JAK3, KIR2DL3, KIR3DL1, KIR3DL2, KLRB1, KLRD1, KLRK1, LIF, MX1, OAS1, OAS2, OAS3, PRF1, PRLR, SIRPA, SPRY4, STAT1, STAT2, TNFSF10	NanoString nsolver Annotation

**Apoptosis**	AKT1, APC, BAD, BAX, BBC3, BCL2, BCL2L1, BCL6B, BID, BIRC3, BIRC5, BLM, CASP1, CASP3, CASP8, CASP9, CD14, CDH1, CTNNB1, FADD, GZMB, HMGB1, LY96, PSMB10, PSMB5, PSMB8, PSMB9, RIPK1, RIPK3, ROCK1, TICAM1, TLR3, TLR4, TNFRSF10B, TNFSF10, TP53	NanoString nsolver Annotation

**Gene Signatures derived in the current study**		
**Gene Expression Signature**	**Genes**	**Reference**
**12 gene- Baseline GES**	BIRC3, CASP9, CD28, CD4, CD5, CTLA4, IL2RB, IRF5, ITGAV, LTB, NFKBIA, RELB	From current study
**31 gene- On-treatment GES**	CXCL3, IRF2, TAPBPL, CYBB, CCL2, CSF1R, MARCO, SYK, IL1B, TLR1, CCL22, TLR8, CCL18, PTPRC, IL10RA, IRF8, IDO1, TLR2, HLA-DMA, ITGAX, IRF1, STAT1, CXCL16, IL15, IL2RA, ICOS, HLA-DRA, CD80, HLA-DPB1, CCL7, CCL8	From current study

<u>Gene annotations for Figure 3E</u>

**S.No**	**Genes**	**Annotation**	**Annotation References**
1.	CXCL9	Interferon gamma signaling	Cao, Y. *et al*[5]
2.	CXCL10	Interferon gamma signaling	Cao, Y. *et al*[5]
3.	CXCL11	Interferon gamma signaling	Cao, Y. *et al*[5]
4.	GBP1	Interferon gamma signaling	Liu, B. *et a l*[6]
5.	GBP2	Interferon gamma signaling	Liu, B. *et al*[6]
6.	GBP4	Interferon gamma signaling	Liu, B. *et al*[6]

7.	IDO1	Interferon gamma signaling	Zhai, L. *et al*[7]
8.	CD3D	T cell activity	Yuan, L. *et al*[8]
9.	NKG7	T cell activity	Li, X. Y. *et al*[9]
10.	TNFRSF9	T cell activity	Sanchez-Paulete, A. R. *et al*[10]
11.	CD28	T cell activity	Alegre, M.-L. *et al* [11]
12.	CXCR6	T cell activity	Mabrouk, N. *et al*[12]
13.	BATF3	T cell activity	Spranger, S. *et al* [13]
14.	RSAD2	T cell activity	Jang, J. S. *et al*[14]
15.	CCL7	Immune cell infiltration	Liu, Y. *et al* [15]<br><br>Zhang, M. *et al*[16]
16.	CCL8	Immune cell infiltration	Yang, P. *et al*[17]
17.	CSF2RB	Immune cell infiltration	Zhu, N. *et al*[18]
18.	LILRB2	Immune cell infiltration	Chen, H. M. *et al*[19]
19.	CTLA4	Immune suppression	Seidel, J. A. *et al* [20]
20.	FOXP3	Immune suppression	Niu, J. *et al*[21]
21.	CXCL13	Immune suppression	Wang, B. *et al* [22]
22.	ENTPD1	Immune suppression	Li, X. Y. *et al*[23]
23.	WDR76	Immune suppression	Ro, E. J. *et al*[24]
24.	CCR4	Immune suppression	Yoshie, O[25]
25.	WNT2	Tumor Progression	Unterleuthner, D. *et al*[26]
26.	COL4A5	Tumor progression	Zeng, X. *et al*[27]
27.	TWIST1	Tumor progression	Li, C. W. *et al*[28]
28.	ICAM5	Tumor progression	Maruya, S. I. *et al*[29]

29.	CSF1	Tumor progression	Fujiwara, T. *et al*[30]
30.	PIK3CG	Tumor progression	Kaneda, M. M. *et al*[31]
31.	GHR	Tumor progression	Basu, R. *et al* [32]
32.	IL11RA	Tumor progression	Johnstone, C. N. *et al* [33]
33.	CLEC14A	Tumor progression	Borah, S. *et al*[34]

Supplementary Table 2: Comparison of anti-PD1 response in TruTumor to other published explant culture platform.

SL#	Reference	Platform	Period of anti-PD1 treatment	Indication	Response phenotypes Observed	TruTumor response phenotypes
1	Voabil, P. et al[35]	Explant histoculture	48h	Melanoma, NSCLC, CaBr, RCC	Cytokine & chemokine analysis show IFN-g, CXCL10 increase in anti-PD1 responding sample	Increased secretion of IFN-g, CXCL9 and CXCL10 in nivolumab responder cohort (SC1) along with gene expression (Fig. 3E & 3F)
					Increased secretion of granzymes and perforins observed in anti-PD1 responding samples	Increased secretion of perforin observed in responder cohort (SC1) while in moderate responder cohort (SC2a) increased secretion of granzyme B was observed (Fig. 3D & Suppl Fig. 6B)
					Low level steady state anti-tumor response of the resident T cells was present in the anti-PD1 responding samples in absence of anti-PD1 treatment.	Baseline gene signature analysis show higher levels of anti-tumor immune activity in responder cohort (SC1) (Fig. 7A).
					Responder samples had less myeloid population	Responders (SC1) had significantly less CD68+ CD206+ macrophage population (Fig. 5A) in untreated arm.
2	Sharon, S. et al[36]	Explant histoculture	24h	HNSCC	Response to anti-PD1 was characterized by increase in secretion of IFN-g and CXCL10	Secretion of IFN-g, CXCL9, CXCL10 increased in Nivolumab responder cohort (SC1) along with gene expression (Fig. 3E & 3F)
3	Yadavilli, S. et al[37]	Explant histoculture	72h	HNSCC	Ex vivo anti-PD1 treatment of tumor tissue significantly increased ICOS gene expression.	ICOS is the part of on-treatment 31 gene expression signature which has higher expression in responder cohort (SC1) compared to non-responder cohort (SC2d) upon Nivolumab treatment (Suppl Fig. 8D)
4	Junk, D. et al[38]	Explant histoculture	72h	Lung	Decrease in tumor fraction was observed in samples responding to anti-PD1 treatment	Nivolumab treatment showed tumor content decrease in responder cohort (SC1) (Fig 3D)

					Spatial analysis of CD8 and PanCK showed an increase in CD8 cells within tumor vicinity in responder but remained unchanged in non-responder	Decrease in distance between CD8 and tumor nest was observed in responder cohort (SC1) (Fig. 4C)

Supplementary Table 3: Antibody details

Antibody	Dilution	Validation	Manufacturer	Catalogue No.	Clone	Assay
CD45 AF700	2:100	Validated in Human peripheral blood lymphocytes	Biolegend	304024	HI30	Flowcytometry
CD3 BV510	3:100	Validated in Human peripheral blood lymphocytes	Biolegend	317332	OKT3	Flowcytometry
CD8 PerCP cy5.5	3:100	Flow cytometric analysis of CD8a expression on human peripheral blood lymphocytes	BD Bioscience	565310	SK1	Flowcytometry
CD4 BV605	3:100	Validated in Human peripheral lymphocytes	Biolegend	317438	OKT4	Flowcytometry
PD1 BV421	3:100	Human peripheral blood lymphocytes were stained with CD3 FITC and CD279 (clone EH12.2H7) Brilliant Violet 421	Biolegend	329920	EH12.2H7	Flowcytometry
CTLA4 APC	5:100	Flow cytometric analysis of CD152/CTLA4 expression on Concanavalin A-activated Human peripheral blood mononuclear cells	BD Bioscience	555855	BNI3	Flowcytometry
CD56 BV711	3:100	flow cytometric analysis of CD56 expression on human peripheral blood lymphocytes	BD Bioscience	563169	NCAM 16	Flowcytometry
CD206 Bv765	3:100	Flow cytometry analysis of GM-CSF stimulated (day3) human peripheral blood monocytes	Biolegend	321142	15-2	Flowcytometry
CD15 BV711	3:100	Human peripheral blood granulocytes were stained with CD15 (SSEA-1, clone W6D3) Brilliant Violet 711	Biolegend	323050	W6D3	Flowcytometry

CD14 perCP cy5.5	3:100	Human peripheral blood monocytes were stained with CD14 (Clone HCD14) PerCP/Cyanine5.5	Biolegend	325622	HCD14/MφP9	Flowcytometry
CD68 PECF594	3:100	Flow cytometric analysis of CD68 expression by human peripheral blood monocytes	BD Bioscience	564944	Y1/82A	Flowcytometry
Foxp3 PECF594	3:100	Flow cytometric analysis of FoxP3 expressed in human lymphocytes	BD Bioscience	562421	259D/C7	Flowcytometry
Ki67 PE	5:100	Profile of Ki-67 PE Set expressed on permeabilized MOLT-4 cell line analyzed by flow cytometry	BD Bioscience	556027	B56	Flowcytometry
GranzymeB PE\Cy7	3:100	Human peripheral blood mononuclear cells were stained with CD8 FITC, fixed, permeabilized, and then stained with Granzyme B	Biolegend	372214	QA16A02	Flowcytometry
panCK AF488	1:100	Flowcytometry analysis for intracellular stain on HeLa cells with pan Cytokeratin Antibody	Novus Biologicals	NBP2-33200	AE-1/AE-3	Flowcytometry
Caspase-3	RTU	FFPE of human Tonsil and Colon tissues.	Biocare	PP229AA	NA	IHC
CD8	RTU	FFPE of human Tonsil and T cell lymphoma	Ventana	7904460	SP57	IHC, mIHC
PD-L1	RTU	FFPE cell lines: NCI-H226 with moderate PD-L1 protein expression and MCF-7 with negative PD-L1 protein expression	Dako	SK006	22C3	IHC

CD4	1:100	FFPE of human Tonsil, Liver, Spleen, Thymoma and Colon tissues.	Abcam	ab133616	EPR6855	mIHC
Foxp3	1:200	FFPE of human Tonsil tissue, Colon, and breast cancer tissue.	Abcam	ab20034	236A/E7	mIHC
pan-CK	RTU	FFPE of human Tonsil	Dako	IR053/IS053	AE1/AE3	mIHC

RTU- Ready to use.

References

1. Foy, J.-P. et al. Datasets for gene expression profiles of head and neck squamous cell carcinoma and lung cancer treated or not by PD1/PD-L1 inhibitors. Data Brief 44, 108556 (2022).
2. Chen, P. L. et al. Analysis of immune signatures in longitudinal tumor samples yields insight into biomarkers of response and mechanisms of resistance to immune checkpoint blockade. Cancer Discov 6, 827–837 (2016).
3. Ayers, M. et al. IFN-γ-related mRNA profile predicts clinical response to PD-1 blockade. *Journal of Clinical Investigation* 127, 2930–2940 (2017).
4. Jorgovanovic, D., Song, M., Wang, L. & Zhang, Y. Roles of IFN-γ in tumor progression and regression: a review. Biomark Res 8, 49 (2020).
5. Cao, Y. et al. CXCL11 Correlates with Antitumor Immunity and an Improved Prognosis in Colon Cancer. Front Cell Dev Biol 9, (2021).
6. Liu, B. et al. Transcriptional Modification and Potential Intracellular Signaling Mechanisms in Human Macrophages Primed by Interferon-γ. Zhongguo Shi Yan Xue Ye Xue Za Zhi 30, 1590–1596 (2022).
7. Zhai, L. et al. IDO1 in cancer: a Gemini of immune checkpoints. Cellular and Molecular Immunology vol. 15 447–457 Preprint at https://doi.org/10.1038/cmi.2017.143 (2018).
8. Yuan, L. et al. CD3D Is an Independent Prognostic Factor and Correlates With Immune Infiltration in Gastric Cancer. Front Oncol 12, (2022).
9. Li, X. Y. et al. NKG7 Is Required for Optimal Antitumor T-cell Immunity. Cancer Immunol Res 10, 154–161 (2022).
10. Sanchez-Paulete, A. R. et al. Deciphering CD137 (4-1BB) signaling in T-cell costimulation for translation into successful cancer immunotherapy. Eur J Immunol 46, 513–522 (2016).
11. Alegre, M.-L., Frauwirth, K. A. & Thompson, C. B. T-cell regulation by CD28 and CTLA-4 Key Points. Nature Reviews Immunology vol. 1(2001).
12. Mabrouk, N. et al. CXCR6 expressing T cells: Functions and role in the control of tumors. Front. Immunol (2022).
13. Spranger, S., Dai, D., Horton, B. & Gajewski, T. F. Tumor-Residing Batf3 Dendritic Cells Are Required for Effector T Cell Trafficking and Adoptive T Cell Therapy. Cancer Cell 31, 711-723.e4 (2017).
14. Jang, J. S. et al. Rsad2 is necessary for mouse dendritic cell maturation via the IRF7-mediated signaling pathway. Cell Death Dis 9, (2018).
15. Liu, Y., Cai, Y., Liu, L., Wu, Y. & Xiong, X. Crucial biological functions of CCL7 in cancer. PeerJ 6, e4928 (2018).
16. Zhang, M. et al. CCL7 recruits cDC1 to promote antitumor immunity and facilitate checkpoint immunotherapy to non-small cell lung cancer. Nat Commun 11, 6119 (2020).
17. Yang, P. et al. Correlation of CCL8 expression with immune cell infiltration of skin cutaneous melanoma: potential as a prognostic indicator and therapeutic pathway. Cancer Cell Int 21, 635 (2021).
18. Zhu, N. et al. CSF2RB Is a Unique Biomarker and Correlated with Immune Infiltrates in Lung Adenocarcinoma. Front Oncol 12, (2022).
19. Chen, H. M. et al. Blocking immunoinhibitory receptor LILRB2 reprograms tumor-associated myeloid cells and promotes antitumor immunity. Journal of Clinical Investigation 128, 5647–5662 (2018).
20. Seidel, J. A., Otsuka, A. & Kabashima, K. Anti-PD-1 and anti-CTLA-4 therapies in cancer: Mechanisms of action, efficacy, and limitations. Frontiers in Oncology vol. 8 Preprint at https://doi.org/10.3389/fonc.2018.00086 (2018).

21. Niu, J. et al. Foxp3 expression in melanoma cells as a possible mechanism of resistance to immune destruction. Cancer Immunology, Immunotherapy 60, 1109–1118 (2011).
22. Wang, B., Wang, M., Ao, D. & Wei, X. CXCL13-CXCR5 axis: Regulation in inflammatory diseases and cancer. Biochimica et Biophysica Acta - Reviews on Cancer vol. 1877 Preprint at https://doi.org/10.1016/j.bbcan.2022.188799 (2022).
23. Li, X. Y. et al. Targeting CD39 in cancer reveals an extracellular ATP-and inflammasome-driven tumor immunity. Cancer Discov 9, 1754–1773 (2019).
24. Ro, E. J. et al. WDR76 degrades RAS and suppresses cancer stem cell activation in colorectal cancer. Cell Communication and Signaling 17, (2019).
25. Yoshie, O. CCR4 as a Therapeutic Target for Cancer Immunotherapy. Cancers (Basel) 13, (2021).
26. Unterleuthner, D. et al. Cancer-associated fibroblast-derived WNT2 increases tumor angiogenesis in colon cancer. Angiogenesis 23, 159–177 (2020).
27. Zeng, X. et al. COL4A family: Potential prognostic biomarkers and therapeutic targets for gastric cancer. Transl Cancer Res 9, 5218–5232 (2020).
28. Li, C. W. et al. Epithelial-mesenchymal transition induced by TNF-α requires NF-κB-mediated transcriptional upregulation of Twist1. Cancer Res 72, 1290–1300 (2012).
29. Maruya, S. I. et al. ICAM-5 (telencephalin) gene expression in head and neck squamous carcinoma tumorigenesis and perineural invasion! Oral Oncol 41, 580–588 (2005).
30. Fujiwara, T. et al. CSF1/CSF1R Signaling Inhibitor Pexidartinib (PLX3397) Reprograms Tumor-Associated Macrophages and Stimulates T-cell Infiltration in the Sarcoma Microenvironment. Mol Cancer Ther 20, 1388–1399 (2021).
31. Kaneda, M. M. et al. PI3Kγ 3 is a molecular switch that controls immune suppression. Nature 539, 437–442 (2016).
32. Basu, R., Wu, S. & Kopchick, J. J. Targeting growth hormone receptor in human melanoma cells attenuates tumor progression and epithelial mesenchymal transition via suppression of multiple oncogenic pathways. www.impactjournals.com/oncotarget (2017).
33. Johnstone, C. N., Chand, A., Putoczki, T. L. & Ernst, M. Emerging roles for IL-11 signaling in cancer development and progression: Focus on breast cancer. Cytokine Growth Factor Rev 26, 489–498 (2015).
34. Borah, S., Vasudevan, D. & Swain, R. K. C-type lectin family XIV members and angiogenesis (Review). Oncol Lett 18, 3954–3962 (2019).
35. Voabil, P. et al. An ex vivo tumor fragment platform to dissect response to PD-1 blockade in cancer. Nat Med 27, 1250–1261 (2021).
36. Sharon, S. et al. Explant Modeling of the Immune Environment of Head and Neck Cancer. Front Oncol 11, (2021).
37. Yadavilli, S. et al. Activating Inducible T-cell Costimulator Yields Antitumor Activity Alone and in Combination with Anti-PD-1 Checkpoint Blockade. Cancer Research Communications 3, 1564–1579 (2023).
38. Junk, D. et al. Human tissue cultures of lung cancer predict patient susceptibility to immune-checkpoint inhibition. Cell Death Discov 7, 264 (2021).
