# Supplementary material for: Tumor histoculture captures the dynamic interactions between tumor and immune components in response to anti-PD1 in head and neck cancer
Source: Nat Commun. 2024 Feb 21;15:1585. doi: 10.1038/s41467-024-45723-z (PMC10881470; doi:10.1038/s41467-024-45723-z)
Supplement: Supplementary file 3 — Reporting Summary [file 41467_2024_45723_MOESM3_ESM.pdf]

nature portfolio

Corresponding author(s): Satish Sankaran

Last updated by author(s): 2024-01-17

Reporting Summary

Nature Portfolio wishes to improve the reproducibility of the work that we publish. This form provides structure for consistency and transparency in reporting. For further information on Nature Portfolio policies, see our Editorial Policies and the Editorial Policy Checklist.

Statistics

For all statistical analyses, confirm that the following items are present in the figure legend, table legend, main text, or Methods section.

n/a	Confirmed	
☐	☒	The exact sample size (*n*) for each experimental group/condition, given as a discrete number and unit of measurement
☐	☒	A statement on whether measurements were taken from distinct samples or whether the same sample was measured repeatedly
☐	☒	The statistical test(s) used AND whether they are one- or two-sided<br>*Only common tests should be described solely by name; describe more complex techniques in the Methods section.*
☐	☒	A description of all covariates tested
☒	☐	A description of any assumptions or corrections, such as tests of normality and adjustment for multiple comparisons
☐	☒	A full description of the statistical parameters including central tendency (e.g. means) or other basic estimates (e.g. regression coefficient) AND variation (e.g. standard deviation) or associated estimates of uncertainty (e.g. confidence intervals)
☐	☒	For null hypothesis testing, the test statistic (e.g. *F*, *t*, *r*) with confidence intervals, effect sizes, degrees of freedom and *P* value noted<br>*Give P values as exact values whenever suitable.*
☒	☐	For Bayesian analysis, information on the choice of priors and Markov chain Monte Carlo settings
☒	☐	For hierarchical and complex designs, identification of the appropriate level for tests and full reporting of outcomes
☒	☐	Estimates of effect sizes (e.g. Cohen's *d*, Pearson's *r*), indicating how they were calculated

Our web collection on statistics for biologists contains articles on many of the points above.

Software and code

Policy information about availability of computer code

Data collection	Imagescope: for viewing whole slide images, Gen5 version 3.03: for viability assay plate reading, BD FACSDiva v8.0.3 for flowcytometry acquisition, Luminex Magpix Exponent 4.2 for cytokine data acquisition, Aperio ScanScope for automated scanning of slides.
Data analysis	GraphPad Prism (Version 9)- for all statistical analysis and graphical representations, Morpheus (https://software.broadinstitute.org/morpheus)- For hierarchical clustering , Watershed Informatics (Boston, USA) for tSNE algorithm, R/Bioconductor package- differential expression analysis and PCA, nSolver 4.0- for NanoString data analysis, QuPath-0.3.0: for multiplex IHC data analysis, Milliplex Analyst v5.1 Flex for converting raw data of cytokine release to pg/ml , FlowJo software (Version 10.8) for flowcytometry analysis, Microsoft 365- Excel

For manuscripts utilizing custom algorithms or software that are central to the research but not yet described in published literature, software must be made available to editors and reviewers. We strongly encourage code deposition in a community repository (e.g. GitHub). See the Nature Portfolio guidelines for submitting code & software for further information.

Data

Policy information about availability of data

All manuscripts must include a data availability statement. This statement should provide the following information, where applicable:

- Accession codes, unique identifiers, or web links for publicly available datasets
- A description of any restrictions on data availability
- For clinical datasets or third party data, please ensure that the statement adheres to our policy

The NanoString data generated in this study are submitted to GEO database (GSE233980: (https://www.ncbi.nlm.nih.gov/geo/query/acc.cgi?acc=GSE233980), [illegible]

GSE234136 (https://www.ncbi.nlm.nih.gov/geo/query/acc.cgi?acc=GSE234136), GSE234138 (https://www.ncbi.nlm.nih.gov/geo/query/acc.cgi?acc=GSE234138)). Publicly available Foy et al 38 clinical data set for CLB-IHN cohort used in this study is available in the GEO database under accession code GSE159067 (https://www.ncbi.nlm.nih.gov/geo/query/acc.cgi?acc=GSE159067). Chen et al 20 clinical data set used in this study was obtained from the manuscript 's Supplementary Tables 1-11 (https://doi.org/10.1158/2159-8290.CD-15-1545). A reporting summary for this article is available as a Supplementary Information file. All the data are available in the article, Supplementary Information and Source Data file.

Research involving human participants, their data, or biological material

Policy information about studies with human participants or human data. See also policy information about sex, gender (identity/presentation), and sexual orientation and race, ethnicity and racism.

Reporting on sex and gender	Consent has been obtained for sharing individual de-identified data. Age and gender information of patients were provided by the collection centers after redacting other personal patient identifiers. Male: 39 (39.8%) and Female 59 (60.2%). Gender based analysis was beyond the scope of this study.
Reporting on race, ethnicity, or other socially relevant groupings	This information was not obtained for this study.
Population characteristics	Age, treatment history , stage of cancer, recurrence status and tumor site information have been reported in this study. (Table 1 and source data file)
Recruitment	Surgical excess tumor and matched blood samples from consented patients with confirmed diagnosis of head and neck squamous cell carcinoma were included in this study. These patients were negative for infectious diseases like hepatitis-B/C, HIV and Covid-19. Contribution of infections towards response to treatment could not be evaluated in this study. Sample was selected based on the availability of the tissue sample and not biased towards any parameters.
Ethics oversight	The study protocol for collection of samples (FCB-PROTOCOL-01) was approved by the Institutional Ethics Committee (IEC) of all the participation centers (Vydehi institute of Medical Sciences & Research Center, DBR & SK Super Speciality Hospital, Mazumdar Shaw Medical Centre, Bangalore Baptist Hospital, Sri Lakshmi Multi Speciality Hospital)

Note that full information on the approval of the study protocol must also be provided in the manuscript.

Field-specific reporting

Please select the one below that is the best fit for your research. If you are not sure, read the appropriate sections before making your selection.

[x] Life sciences [ ] Behavioural & social sciences [ ] Ecological, evolutionary & environmental sciences

For a reference copy of the document with all sections, see nature.com/documents/nr-reporting-summary-flat.pdf

Life sciences study design

All studies must disclose on these points even when the disclosure is negative.

Sample size	No sample size calculation was performed. Based on the response rate (15-20%) of Nivolumab in HNSCC, sample size of 50 is sufficient enough to evaluate the response.
Data exclusions	Post sample qualification all samples underwent histopathology, viability assay and cytokine profiling. For other assays, insufficient sample quantity restricted data generation.
Replication	All data presented in this study had biological replicates.
Randomization	Experimental grouping was not relevant for the study as all samples underwent Nivolumab treatment (Figure1 B)
Blinding	Data collection and analysis was performed blinded using coded treatment arm names.

Reporting for specific materials, systems and methods

We require information from authors about some types of materials, experimental systems and methods used in many studies. Here, indicate whether each material, system or method listed is relevant to your study. If you are not sure if a list item applies to your research, read the appropriate section before selecting a response.

Materials & experimental systems

n/a	Involved in the study	
[ ]	[x]	Antibodies
[ ]	[x]	Eukaryotic cell lines
[x]	[ ]	Palaeontology and archaeology
[x]	[ ]	Animals and other organisms
[x]	[ ]	Clinical data
[x]	[ ]	Dual use research of concern
[x]	[ ]	Plants

Methods

n/a	Involved in the study	
[x]	[ ]	ChIP-seq
[ ]	[x]	Flow cytometry
[x]	[ ]	MRI-based neuroimaging

Antibodies

Antibodies used

IHC or mIHC: Caspase-3,Biocare,APR229AA,Rabbit Polyclonal,041023A; Fox-P3,Abcam,ab20034,236A/E7,GR3189545-1; CD4,Abcam,ab133616,EPR6855,GR3276764-14; CD8,Ventana,790-4460,SP57,H20504; panCK,Dako,IS053,AE1/AE3,10149464; PDL-1,Dako,SK006,22C3,10149522
Flowcytometry: CD45: AF700,Biolegend,304024 ,HI30,B349930; CD3: BV510,Biolegend,317332 ,OKT3,B340677; CD8: perCP Cy5.5,BD Bioscience,565310,SK1,1040337; CD4: BV605,Biolegend,317438,OKT4,B350846; PD1: BV421,Biolegend,329920,EH12.2H7,B336297; CTLA4: APC,BD Bioscience,555855,BNI3,335549; CD56: BV711,BD Bioscience,563169 ,NCAM 16,184750; CD206: BV785,Biolegend,321142,15-2,B334359; CD15: BV711,Biolegend,323050,W6D3,B324659; CD14: perCP Cy5.5,Biolegend,325622,HCD14/MϕP9,B350814; CD68: PE-CF594,BD Bioscience, 564944 ,Y1/82A,1243837; Foxp3: PE-CF594,BD Bioscience,562421 ,259D/C7,1250304; Ki67: PE,BD Bioscience,556027,B56,6263616; GranzymeB: PE\Cy7,Biolegend, 372214,QA16A02,B330885; panCK: AF488,Novus Biologicals,NBP2-33200,AE-1/AE-3,040121-AF488

Antibodies used is provided as supplementary table 3 in the manuscript

Validation

The antibodies used for IHC was recommended for IHC use for human species and validated in the recommended positive control by manufacturer and HSNCC samples. The antibodies and the manufacturer validation tissue/statement are as follows:
Caspase-3:Colon cancer
Fox-P3:Tonsil
CD4:Lymph Node
CD8:Tonsil/Lymph Node
panCK:Tonsil
PDL-1:PD-L1 testing with PD-L1 IHC 22C3 pharmDx was used to assess PD-L1 expression in patients with metastatic or unresectable recurrent HNSCC in the KEYNOTE-048 clinical trial

For antibodies used in Flocytometry was recommended for flowcytometry and human species. The antibodies and the manufacturer validation statement are as follows:
CD45: Validated in Human peripheral blood lymphocytes
CD3: Validated in Human peripheral blood lymphocytes
CD8: Flow cytometric analysis of CD8a expression on human peripheral blood lymphocytes
CD4: Validated in Human peripheral lymphocytes
PD1: Human peripheral blood lymphocytes were stained with CD3 FITC and CD279 (clone EH12.2H7) Brilliant Violet 421
CTLA4: Flow cytometric analysis of CD152/CTLA4 expression on Concanavalin A-activated Human peripheral blood mononuclear cells
CD56: flow cytometric analysis of CD56 expression on human peripheral blood lymphocytes
CD206: Flow cytometry analysis of GM-CSF stimulated (day3) human peripheral blood monocytes
CD15: Human peripheral blood granulocytes were stained with CD15 (SSEA-1, clone W6D3) Brilliant Violet 711
CD14: Human peripheral blood monocytes were stained with CD14 (Clone HCD14) PerCP/Cyanine5.5
CD68: Flow cytometric analysis of CD68 expression by human peripheral blood monocytes
Foxp3: Flow cytometric analysis of FoxP3 expressed in human lymphocytes
Ki67: Profile of Ki-67 PE Set expressed on permeabilized MOLT-4 cell line analyzed by flow cytometry
GranzymeB: Human peripheral blood mononuclear cells were stained with CD8 FITC, fixed, permeabilized, and then stained with Granzyme B
panCK: Flowcytometry analysis for intracellular stain on HeLa cells with pan Cytokeratin Antibody

Validation details are provided in the supplementary table 3 in the manuscript

Eukaryotic cell lines

Policy information about cell lines and Sex and Gender in Research

Cell line source(s)

Cell Line was procured from NCCS, Pune, India

Authentication

The cell lines available in NCCS were authenticated using Short Tandem Repeat (STR) analysis

Mycoplasma contamination

Report from NCCS stated that the HeLa cell line tested negative for Mycoplasma

Commonly misidentified lines
(See ICLAC register)

Name any commonly misidentified cell lines used in the study and provide a rationale for their use.

Flow Cytometry

Plots

Confirm that:

☒ The axis labels state the marker and fluorochrome used (e.g. CD4-FITC).

☒ The axis scales are clearly visible. Include numbers along axes only for bottom left plot of group (a 'group' is an analysis of identical markers).

☒ All plots are contour plots with outliers or pseudocolor plots.

☒ A numerical value for number of cells or percentage (with statistics) is provided.

Methodology

Sample preparation	Single cells were prepared from tumor explants as per MACS Miltenyi tumor dissociation protocol using three enzyme combinations, including Enzyme H, EnzymeR, and Enzyme A.
Instrument	BD LSRFortessa Cell Analyzer : 649225B9 - LSRFORTESSA 6B/4R/6V/2UV 4 LASER
Software	BD FACSDiva v8.0.3
Cell population abundance	No sorting was performed
Gating strategy	Lineage gating strategy: Live->Cell->Single cell using FSC-> Single cell using SSC-> CD45+-> CD45+ Low SSC-> Low SSC CD3+ -> CD3+ CD8+ and CD4+ --> CD4+FoxP3+. Low SSC CD3- --> CD56+ and CD56-. CD45+High SSC -> CD14+, CD68+ --> CD68+CD206+, CD15+. Functional markers were gated for relevant cell type. Lymphocyte population was used as the lower limit for gating for cells using FSC/SSC. For markers unstained cells and FMO based gating was performed.

☒ Tick this box to confirm that a figure exemplifying the gating strategy is provided in the Supplementary Information.
